# Author reply to McDonough

**DOI:** 10.1111/imj.70040

**Published:** 2025-04-01

**Authors:** Brendan J. Nolan, Ada S. Cheung

**Affiliations:** ^1^ Trans Health Research Group, Department of Medicine (Austin Health) University of Melbourne Melbourne Victoria Australia; ^2^ Department of Diabetes and Endocrinology Princess Alexandra Hospital Brisbane Queensland Australia; ^3^ Equinox Gender Diverse Clinic Thorne Harbour Health Melbourne Victoria Australia; ^4^ Department of Endocrinology Austin Health Melbourne Victoria Australia

We appreciate McDonough's^1^ letter but note that it contains frequently debunked disinformation.^2^ Our Clinical Perspective article ‘Gender affirming hormone therapy for transgender and gender‐diverse adults in Australia’ provides recommendations for the initiation and monitoring of gender‐affirming hormone therapy (GAHT) in transgender and gender‐diverse (trans) adults.^3^ Despite this, McDonough raises concerns about trans children and youth,^1^ which is beyond our article's scope. Furthermore, our article is unrelated to the Cass review of gender identity services for children in the United Kingdom, a flawed and unbalanced report inconsistent with established global standards.^4^

Gender‐affirming care is an evidence‐informed, patient‐centred approach that supports trans individuals in their personal goals of better aligning their physical, psychological and social characteristics with their gender identity.^5^ It has the goals of improving mental health, reducing gender dysphoria and enhancing overall quality of life, while respecting individual values and bodily autonomy. In contrast to simply ‘accepting the subjective interpretation of gender by a young person’, gender‐affirming care involves comprehensive evaluation of coexisting mental health conditions and facilitating self‐exploration of identity and gender, as well as social and psychological support.^5^ Some but not all trans individuals request GAHT and/or surgical interventions. Notably, these interventions are associated with improvements in mental health, including reductions in gender dysphoria, depression and suicidality in trans adults^6^, ^7^ and youth.^8^

McDonough's claim that the World Professional Association for Transgender Health (WPATH) is ‘discredited’ relies on a non‐peer‐reviewed source from a conservative Christian lobby group. Gender‐affirming care is supported by not only WPATH but also over 30 professional medical associations, including the Australian Medical Association and the Royal Australasian College of Physicians.^9^, ^10^, ^11^

Similarly, the notion of social contagion or rapid‐onset gender dysphoria is unsubstantiated. The only study suggesting it, based on parental reports rather than direct engagement with trans youth, has been widely criticised and corrected.^12^ Population data contradict this theory, showing stabilisation of the number of people identifying as trans^13^ and stable proportions of gender dysphoria diagnoses over time.^14^

Restricting gender‐affirming care or waiting to find the ‘cause’, as proposed by McDonough, is unethical and harmful and completely disregards the lived experiences and needs of trans individuals, who are not a ‘new disease phenomenon’. Such restrictions increase risks of suicide, depression, anxiety, disordered eating and a diminished quality of life.^8^, ^15^, ^16^, ^17^, ^18^, ^19^, ^20^

Gender incongruence is not a disorder, nor is it a condition of mental ill health, and it is not comparable to opioid use disorder. Sex steroid formulations used in GAHT are identical to those prescribed to cisgender individuals for the treatment of hypogonadism or menopause and are neither addictive nor harmful.^21^

The gender‐affirming model of care is supported by multiple studies summarised in our review and here.^2^, ^8^, ^15^, ^16^, ^17^, ^18^, ^19^ Evidentiary standards for gender‐affirming care should not be subjected to standards that are not applied elsewhere in medicine.^4^

As for any treatment in medicine, a shared decision‐making approach carefully balancing potential benefits with potential risks should be taken, involving patients, their family, and their treating clinician. Medical interventions should be guided by scientific evidence, clinical expertise and patient‐centred care in accordance with established guidelines from reputable medical organisations, free from political interference. Most importantly, the voices and perspectives of trans individuals must be recognised and respected, rather than dehumanised by efforts to impose rigid gender binaries and norms driven by conservative ideologies.
